# Aspiration of the Bowels in Peritonitis

**Published:** 1882-03

**Authors:** 


### ASPIRATION OF THE BOWELS IN PERITONITIS.

A successful instance of this measure is reported by Dr. D. M. Williams, in the Dublin *Journal of Medical Science*. The patient was a boy of thirteen. We quote the most interesting part of the history :—

His condition was now alarming ; the pulse was, for the first time, irregular and compressible—144 to the minute ; breathing very shallow ; eyes sunken ; cheeks hollow ; tongue dry ; constantly moaning with pain—evidently dying. He placed his hand on the epigastrium, and said the pain was smothering him, no doubt from pressure upward of the diaphragm interfering with the action of the heart and lungs. The abdomen was arched from the xiphoid appendix to pubes, the least attempt at percussion causing great agony. Had not passed water since the 7th. I determined to aspirate him, and passed the finest needle into the tranverse colon, and on turning the tap a great quantity of flatus rushed through, followed by three ounces of fluid fæces, which gave him great relief, but did not perceptibly diminish the size of the abdomen. Fearing the needle was blocked, I withdrew it, and found such was not the case. I had evidently emptied this portion of the colon. Having washed the needle, I pierced the ascending colon ; another rush of flatus took place, followed by eight ounces of fæces. I repeated the operation on the descending colon, with the same results. There was now very decided diminution of distention and relief of pain : still he complained bitterly of a spot just below the navel, which was quite tympanitic. Taking care to avoid the bladder, I pierced probably the ileum ; more flatus escaped, with about half an ounce of fluid fæces. He was now much relieved ; pulse had fallen to 96 ; breathed deeper.

10 P. M. Much the same as after the tapping; expression of face less haggard; pulse 120, full and soft: temperature 102°; passed water freely, and without pain, an hour after the tapping. To take pulv. Doveri, gr. 10, h. s. From this time his progress toward recovery was steady.

